# Implementation of a Patient Summary Web Application According to the International Patient Summary and Validation in Common Use Cases in Japan

**DOI:** 10.1007/s10916-023-01993-6

**Published:** 2023-09-23

**Authors:** Chong Song, Masaharu Nakayama

**Affiliations:** Department of Medical Informatics, Tohoku University Graduate School of Medicine, 2-1 Seiryo-machi, Aoba-ku, Sendai, 980-8574 Miyagi Japan

**Keywords:** International Patient Summary, Discharge summary, Electronic health record, Fast health interoperability resource, Web application, Interactive visualization

## Abstract

**Background:**

The application of standardized patient summaries would reduce the risk of information overload and related problems for physicians and nurses. Although the International Patient Summary (IPS) standard has been developed, disseminating its applications has challenges, including data conversion of existing systems and development of application matching with common use cases in Japan. This study aimed to develop a patient summary application that summarizes and visualizes patient information accumulated by existing systems.

**Methods:**

We converted clinical data from the Standardized Structured Medical Information eXchange version 2 (SS-MIX2) storage at Tohoku University Hospital into the Health Level 7 Fast Healthcare Interoperability Resource (FHIR) repository. Subsequently, we implemented a patient summary web application concerning the IPS and evaluated 12 common use cases of the discharge summary.

**Results:**

The FHIR resources of seven of the necessary IPS sections were successfully converted from existing SS-MIX2 data. In the main view of the application we developed, all the minimum necessary patient information was summarized and visualized. All types of mandatory or required sections in the IPS and all structured information items of the discharge summary were displayed. Of the discharge summary, 75% of sections and 61.7% of information items were completely displayed, matching 12 common use cases in Japan.

**Conclusions:**

We implemented a patient summary application that summarizes and visualizes patient information accumulated by existing systems and is evaluated in common use cases in Japan. Efficient sharing of the minimum necessary patient information for physicians is expected to reduce information overload, workload, and burnout.

## Background

The increase in electronic health records (EHRs) is supposed to improve care quality, efficiency, and safety, increase patient engagement, and reduce healthcare disparities [1, 2]. With the spread of EHRs and health information exchange (HIE), long-term and wide-ranging data over a patient’s lifetime have been accumulated [3]. When using shared patient information between different hospitals and clinics to treat patients, physicians and nurses must obtain useful patient information in a limited time [4]. Summarizing important patient information is difficult, and the amount of clinical data leads to information overload [5]. As information overload occurs when increasing but poorly organized information is received, better organized and summarized information would help reduce the workload of physicians and nurses in searching for and processing patient data [1]. As the possible effects of information overload include failing to process some of the inputs and giving up the search for needed information, a simple view of the minimum required patient information would be helpful for a quick look at the patient data in a limited time [6].

The International Patient Summary (IPS) is a standard for summarizing the minimum required patient information [7]. An IPS document is an EHR extract containing a patient’s essential healthcare information to support the use case scenario, such as unplanned cross-border care. In the IPS Implementation Guide, 14 sections (including medication summary and problem list) were divided into three priority groups (Required, Recommended, and Optional) [8]. In the International Organization for Standardization (ISO) standard, 19 sections and collections are also divided into five conformance groups (Mandatory, Required, Required if known, Conditional, and Optional) [9].

This study aimed to develop a patient summary application that summarizes and visualizes patient information accumulated by EHRs and HIE to efficiently share the minimum necessary patient information between physicians and nurses in different hospitals in Japan. However, there are issues with applying IPS in Japan. The first issue is mapping clinical data in Standardized Structured Medical Information eXchange 2 (SS-MIX2), a standard clinical data storage system in Japan, to Health Level 7 Fast Healthcare Interoperability Resource (HL7 FHIR) [10]. Second, it is necessary to confirm whether the IPS can be fully utilized in domestic use cases in Japan and to conduct user experience (UX) testing.

## Materials and Methods

**Fig. 1:**
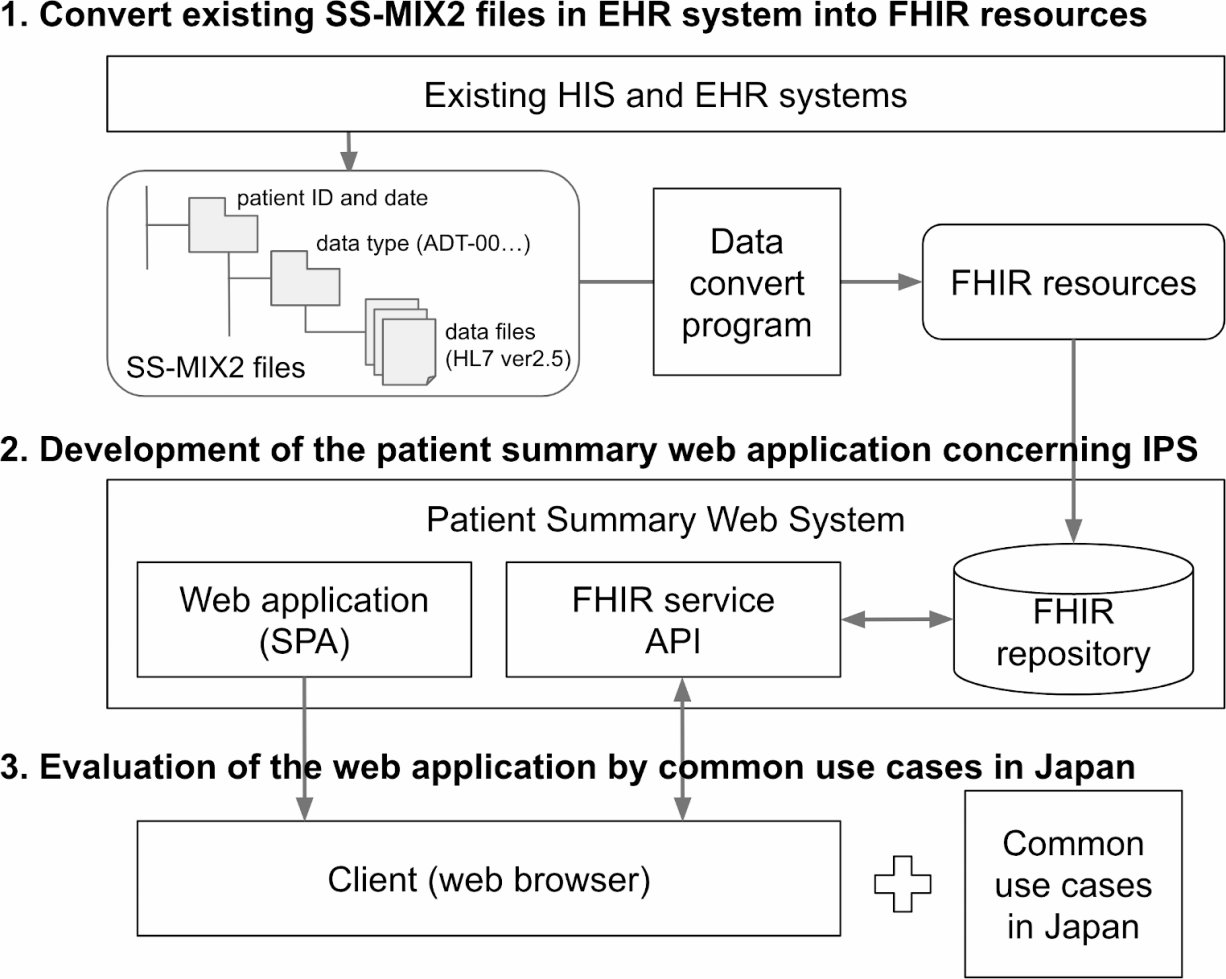
Overview of materials and methods

This study proceeded in three main steps (Fig. 1). First, we acquired patient data from the SS-MIX2 standardized storage and converted it into FHIR resources using a conversion tool independently developed in our previous study [12]. The FHIR resources of patient data were stored in the FHIR repository. Second, we extracted the minimum necessary information items from the IPS standardization and developed a patient summary web application that summarizes and visualizes the necessary minimum information items. Finally, we evaluated this web application based on common use cases in Japan. The following three sections provide details.

### Convert Existing SS-MIX2 Files in the EHR System into FHIR Resources

The format defined by SS-MIX2 is a standardized format that complies with HL7 version 2.5 and various regulations of the Japanese Association of Healthcare Information Systems Industry [11]. Existing Hospital Information System (HIS) and EHR systems output data into SS-MIX2 standardized storage whenever a transaction occurs. The data in SS-MIX2 standardized storage are stored in folders for each data type in the format of HL7 version 2.5 files. As SS-MIX2 aims to improve medical care quality by exchanging and sharing medical information for all medical institutions in Japan, the SS-MIX2 specification extracts from the HL7 version 2.5 specify some data segments commonly used for data exchange between systems in Japan. Because data are outputted for each transaction in SS-MIX2, it is necessary to convert chronologically arranged HL7 version 2.5 messages into FHIR resources.

Our previous study demonstrated a trial of conversion from SS-MIX to FHIR resources. We exported the data of dummy patients in the existing HIS and EHR systems at Tohoku University Hospital into the SS-MIX2 format [12]. In this research, we used data from dummy patients to convert the minimum necessary information items from SS-MIX2 to the FHIR format. We prepared dummy patient data with all information items, which were more suitable for this research, without unnecessary privacy issues. We also referred to the HL7 FHIR JP Core Implementation Guide Draft version 1 (JPcore) when converting FHIR resources [13]. As the suggested code system in Japan is slightly different from the IPS, we applied the code systems mentioned in JP core. Our data conversion process, which transforms SS-MIX2 data into FHIR resources, was conducted using a mapping table and pipeline that we developed in a previous study [12]. This process involves applying the code systems from JP core to our mapping table and then transforming the data into a FHIR repository server. The mapping pipeline is based on InterSystems IRIS (Cambridge, MA, USA).

### Development of the Patient Summary Web Application Concerning IPS

We extracted all the Required and Recommended sections in the IPS Implementation Guide as well as all the Mandatory, Required, and Required if known Sections and Attribute collections in the IPS ISO document [9]. In this study, ten section items labeled as Mandatory, Required, and Required if known were in the IPS ISO selected as the “minimum necessary patient information.“ We then acquired this information from the converted FHIR resources and implemented it in the patient summary application. To improve the efficiency of information collection, we attempted to display the minimum necessary patient information from one view and avoid the burden of information retrieval. We implemented several features, such as Encounter List, Timeline, and Filter & Search, to efficiently display point-in-time information from one view of the application.

We used the Firely Server R4 [14] as the FHIR server and SQLite [15] as the FHIR database. Because the web-based EHR system has become popular, we implemented the patient summary application as a web application. As some interactive features such as Timeline and Filter & Search were required, we implemented it as a single-page application using the Nuxt.js framework [16] and JavaScript. We used the FHIR Representational State Transfer application programming interface and JavaScript Object Notation formats for data exchange between the web application and the FHIR server.

### Evaluation of the Web Application by Common Use Cases in Japan

Standardized specifications for overall patient summaries have not yet been established in Japan. Whether the IPS matches Japanese local use cases is unclear. In contrast, discharge summaries have been commonly used and standardized in Japan. A discharge summary summarizes and aggregates patient health information at discharge. It is widely available in hospitals and clinics in Japan for effective communication with other medical staff [17]. The Guidance on Creating Discharge Summary [18] and Discharge Summary Terms Based on HL7 Clinical Document Architecture Release 2 [19] were published in 2019. However, the Discharge Summary HL7 FHIR Description Specification (DSDS) First Edition was released in 2021 [20]. The patient summary application was evaluated based on the Discharge Summary.

We extracted all mandatory sections and parts of the optional sections described in the DSDS First Edition and mapped them with the IPS sections. We then compared these sections with the information displayed on the application to confirm whether the data of the mandatory sections can be displayed on the main views of the application.

We extracted all information items (e.g., patient attributes, current problems, and everyday medicine) from 12 descriptive examples in the Guidance on Creating Discharge Summary. We tested 12 descriptive examples (i.e., cardiology, gastroenterology, general medicine, urology, obstetrics, gynecology, neurology, gastroenterology, pediatrics, orthopedics, orthopedics rehearsal, and psychosomatic medicine) as common use cases. We developed features in the application, such as the Timeline, Encounter List, and Filter & Search, to show the information items corresponding to the common use cases in the main view of the application.

## Results

### Minimum Necessary Patient Information Acquired from Existing Systems

We extracted all mandatory or required sections from the IPS ISO standard and the IPS Implementation Guide as the minimum necessary patient information. Furthermore, we converted all available patient information from the SS-MIX2 data of dummy patients into FHIR resources. As a result, among the 10 types of necessary information, 7 were converted from SS-MIX2 data. Because the other three recommended sections needed to be obtained from the SS-MIX2 conversion data, we implemented these three sections directly from POST and GET FHIR resources from the FHIR server. Finally, all 10 sections regarding the minimum necessary patient information were available in the web application (Table 1). In Table 1, items show the number of minimum necessary items count / all items count. For example, in Patient Attributes, the conformance of seven items, such as Patient’s name, Patient’s address and telecom, Administrative gender, Date of birth, Healthcare related identifiers, Patient identifier, and insurance identifier, are Mandatory, Required, or Required if known.

**Table 1:** Data mapping for minimum necessary patient information

#	IPS sections	IPS ISO ^a^	IPS IG ^b^	Items ^c^	Data source	FHIR resources
1	Patient Attributes	M	H	7/11	SS-MIX2	Patient
2	Healthcare Provider and Patient’s Address Book	RK	H	4/7 6/8	SS-MIX2	Practitioner, Organization
3	Provenance Metadata	M	H	9/10	SS-MIX2	Practitioner, Organization
4	Allergies and Intolerances	M	R	10/17	SS-MIX2	AllergyIntolerance
5	Problems	M	R	6/10	SS-MIX2	Condition
6	Medication Summary	M	R	13/20	SS-MIX2	MedicationRequest
7	Results/Observed condition	RK	RC	6/9	SS-MIX2	Observation
8	History of Procedures	RK	RC	4/7	Other	Procedure
9	Medical Devices	RK	RC	4/7	Other	DeviceUseStatement
10	Immunizations	RK	RC	5/12	Other	Immunization

^a^ IPS ISO standard; *M* Mandatory, *RK* Required if known

^b^ IPS Implementation Guide; *H* Header, *R* Required, *RC* Recommended

^c^ Items count of section in IPS ISO standard; minimum necessary items count / all items count

### Minimum Necessary Patient Information Displayed on One Main View

**Fig. 2:**
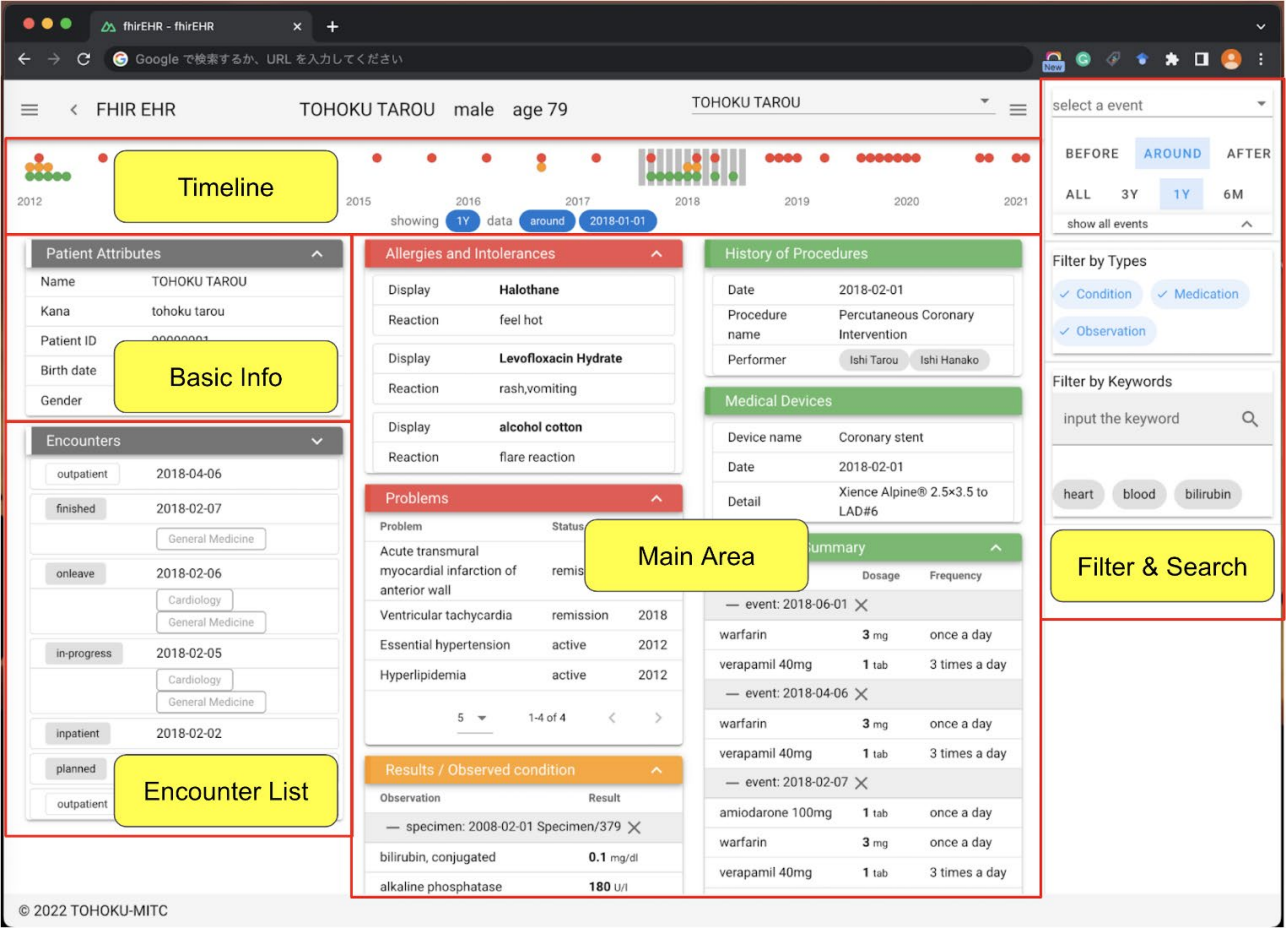
One main view of the patient summary web application

We developed a web application that uses FHIR resources converted from SS-MIX2 standard storage to summarize and visualize patient information. Additionally, the application displays the minimum necessary patient information briefly on one main view (Fig. 2). The one main view of the patient summary application is mainly divided into three parts: Timeline, Main Area, and Filter & Search features. In the Main Area, minimum necessary patient information was displayed in each section. Patient Attributes, Allergies and Intolerances, History of Procedures, and Medical Devices are vital information that does not change much and are thus on the left side. Problems and Encounters are displayed in the middle. As the Observed condition and Medication Summary could be changed due to period and use case, they are on the right side, near the Filter & Search feature.

**Fig. 3:**
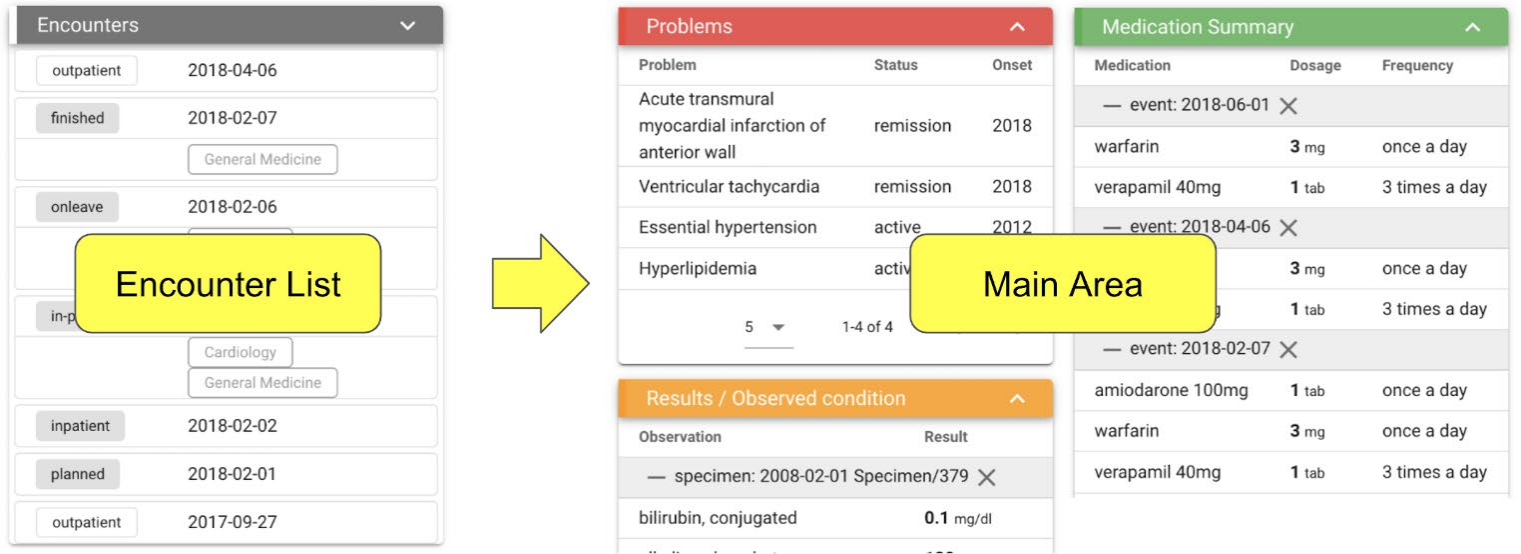
Encounter List feature of the patient summary web application

We implemented an Encounter List feature to display the necessary patient information according to the timing of the encounter event (Fig. 3). Encounter events can be arranged in descending order of date. Furthermore, data such as dates and institutions are displayed for each event type, including outpatient, inpatient, and discharge. The Encounter List supports switching between the full and summary list views. In the full-list view, the events are shown individually. In the summary list view, events from one inpatient to discharge are displayed together as one event, and outpatient events between two inpatient events are displayed alongside the number of events. Patient information was filtered based on the date of the encounter event. Use cases like showing a single hospital admission or visit would be achieved this way.

**Fig. 4:**
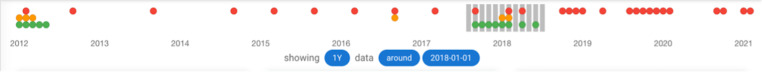
Timeline feature of the patient summary web application

Furthermore, a Timeline feature was implemented to provide an overview of the patient’s lifelong information (Fig. 4). The horizontal axis of the timeline represents years and months. Additionally, the events are organized by month and information type. If there was an event of interest, the dot was plotted. The red dots represent problems, the orange dots represent diagnostic results, and the green dots represent prescription events. The gray part is the data display period, which can be changed by clicking on the timeline. Use cases such as showing the information during admission and discharge would be achieved this way.

Most use cases would be achieved using the Encounter List and Timeline features. However, there would be more complex situations, such as filtering all the information on some diseases. A Filter & Search feature was implemented for this type of use. The Filter & Search feature filters patient information by period, event type, and keywords. Finally, we could display all 10 sections of the minimum necessary patient information from the main view.

### Evaluation of the Application by Common Use Cases in Japan

We evaluated the patient summary application based on the Discharge Summary (DS), which included Japan’s most popular use cases. First, we extracted 16 sections from the Discharge Summary HL7 FHIR Description Specifications (DSDS) and mapped them onto the IPS sections. In the application, 12 (75%) of the 16 sections of the DSDS were displayed. Second, the six mandatory sections in both the DS and IPS are displayed in the application.

We extracted all 20 information items from 12 descriptive examples described in the Guidance on Creating Discharge Summary (GCDS). These 20 items can be divided into three priority groups (Mandatory, Recommended, and Optional) or two categories (structured and unstructured narrative information). We confirmed that our application functionally summarized and visualized patient information based on 12 commonly used cases (Table 2). The 12 use cases are examples of major medical departments in Japan, and while some items are common to each of the use cases, others are unique in specific cases. Information items not included in individual use cases were considered invalid and marked by “ー”. Information items completely displayed on the application were marked by “◯”, and absent or incompletely displayed items were marked by “△”. Except for invalid items, each use case contains 16.3 items, and 10.1 items were displayed on average. In all 196 information items of the 12 use cases, 121 (61.7%) were displayed. The absent or incompletely displayed items were mainly unstructured narrative information, which was not included in SS-MIX2 storage. All 81 structured items of the 12 use cases are displayed. In contrast, in 115 unstructured narrative items, only 40 (34.8%) were displayed.

**Table 2:** Items in 12 use cases of the Discharge Summary displayed on the application

			Twelve Use Cases in the Discharge Summary ^b^											
The GCDS Items ^a^			1	2	3	4	5	6	7	8	9	10	11	12
Patient Attributes	M	S	◯	◯	◯	◯	◯	◯	◯	◯	◯	◯	◯	◯
Provenance Metadata	M	S	◯	◯	◯	◯	◯	◯	◯	◯	◯	◯	◯	◯
Encounter	M	S	◯	◯	◯	◯	◯	◯	◯	◯	◯	◯	◯	◯
Allergies and Intolerances	M	S	◯	◯	◯	◯	◯	◯	◯	◯	◯	◯	◯	◯
Devices	R	S	◯	ー	ー	◯	ー	ー	ー	ー	ー	ー	ー	ー
Problems on discharge ^c^	M	S	◯	◯	◯	◯	◯	◯	◯	◯	◯	◯	◯	◯
Inpatient Reasons ^d^	M	N	△	△	△	△	△	△	△	△	△	△	△	△
Current Problems ^e^	O	N	◯	◯	◯	◯	◯	◯	◯	◯	◯	◯	◯	◯
History of Past Problems ^f^	O	N	◯	◯	◯	◯	◯	◯	ー	◯	◯	◯	◯	◯
Lifestyle/Habit ^g^	O	N	△	ー	△	△	ー	ー	△	△	ー	△	△	△
Family History ^h^	O	N	△	ー	△	△	ー	ー	ー	△	ー	△	△	△
Everyday Medicine ^i^	O	N	ー	◯	◯	◯	ー	ー	ー	◯	ー	◯	◯	◯
Physical Findings ^j^	O	N	ー	△	△	△	△	△	△	△	△	△	△	△
Diagnostic Results ^k^	O	N	◯	◯	◯	◯	ー	◯	ー	◯	◯	◯	◯	◯
Nursing Summary ^l^	M	N	△	△	△	△	△	△	△	△	△	△	△	△
History of Procedures	R	S	◯	ー	ー	◯	◯	◯	ー	◯	ー	ー	△	ー
Immunizations	M	S	ー	ー	ー	ー	ー	ー	ー	ー	ー	ー	ー	◯
Discharge Status ^m^	M	N	△	△	△	△	△	△	△	△	△	△	△	△
Medication on discharge ^n^	M	S	◯	◯	◯	◯	◯	◯	◯	◯	◯	◯	◯	◯
Plan of Care ^o^	M	N	△	△	△	△	△	△	△	△	△	△	△	△

^a^ Guidance on Creating Discharge Summary; *M* Mandatory, *R* Recommended, *O* Optional, *S* Structured, *N* Narrative

^b^ Twelve use cases in the Discharge Summary; *1* cardiology, *2* gastroenterology, *3* general medicine, *4* urology, *5* obstetrics, *6* gynecology, *7* neurology, *8* digestive surgery, *9* pediatrics, *10* orthopedics, *11* orthopedic rehearsal, *12* psychosomatic medicine

Results of items on application: *ー* item not included in the use case, ◯ item displayed, △ item absent or incompletely displayed

c Problems on discharge; diagnosis at discharge, indicated in the Problems section

d Inpatient Reasons; chief complaint or reason for admission to the hospital, to be stated in the text

e Current Problems; history of current illnesses, main diseases, and pathological conditions leading to admission and the decision on the indications for admission, displayed in the Problems section

f History of Past Problems; history of past problems, displayed in the Problems section

g Lifestyle/Habit; preferences, particularly smoking and drinking history

h Family History; family history information considered necessary for the patient’s health care

i Everyday Medicine; information on medications, displayed in the Medication Summary section

j Physical Findings; physical findings on admission

k Diagnostic Results; admission laboratory results, displayed in the Results/Observed condition section

l Nursing Summary; Admission progress, a textual brief description of hospitalization progress

m Discharge Status; status at discharge, the text describes status at discharge

n Medication on discharge; information on medications used at discharge, displayed in the Medication Summary section

o Plan of Care; discharge policy, the text describes discharge policy

**Fig. 5:**
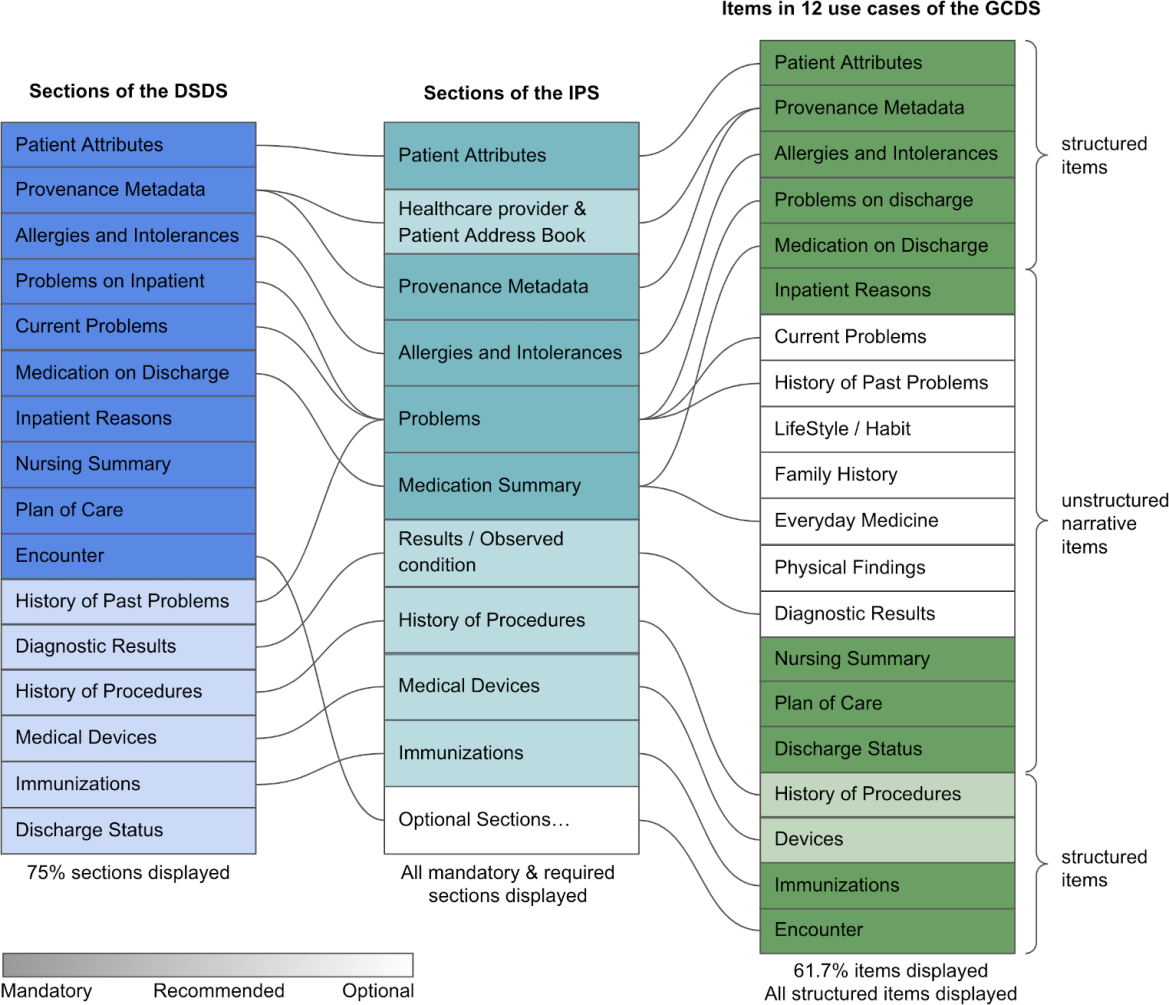
Sections and items mapping between the IPS and the Discharge Summary

Sections and item mapping between the IPS and the Discharge Summary are shown in Fig. 5. We extracted all 10 mandatory or recommended sections from the IPS as the minimum necessary patient information. Subsequently, we implemented them all in the application. These 10 sections were mapped to 12 sections of the Discharge Summary. Furthermore, in all 16 sections of the Discharge Summary, 12 (75%) were displayed on the application. Furthermore, as these 10 sections were mapped to the information items in 12 common use cases of the Discharge Summary, 61.7% of the items, including all structured items, were displayed on the application.

## Discussion

This study developed a patient summary application concerning IPS using FHIR resources converted from data in SS-MIX2 standardized storage. To the best of our knowledge, this is the first study to develop an application using FHIR data converted from SS-MIX2. All critical sections that are mandatory for the IPS and discharge summary were handled. After confirming the use cases of the Japanese discharge summary, the most important items and features necessary for on-site application were generally supported. We verified the feasibility of developing a patient summary application concerning IPS that summarizes and shares the minimum indispensable patient information between physicians and nurses in different hospitals in Japan. Additionally, we confirmed the issues to be solved before practical use. We consider that developing an application that does not exhaustively display many information items, but rather simply consolidates the minimum necessary information regarding the IPS standard on a single screen, has the following two advantages: (1) Versatility, as this application seamlessly integrates various clinical data with interoperability based on international standards regardless of the use cases, and (2) Convenience, as patient information to be shared can be efficiently confirmed on a single user-friendly screen in a short time. These features play a pivotal role in enhancing healthcare by addressing essential information needs for both medical staff and patients.

Since data are outputted for each transaction in SS-MIX2, inpatient and discharge are not linked in the data source. We converted each SS-MIX2 file of an encounter event into one FHIR Encounter resource. Consequently, we implemented a feature to connect events during a programmable pair of inpatient and discharge periods. This feature works well when both admission and discharge event data are available. However, it became a problem because of missing data. A fundamental solution would be to output explicit linking relationships between admission and discharge into SS-MIX2 data.

“Recommended” items in the IPS, such as History of Procedures, Devices, and Immunizations, could not be obtained from SS-MIX2 data. These features were implemented by directly registering FHIR resources instead of using the SS-MIX2 data source. The SS-MIX2 specification extracts only partial data segments commonly used for data exchange between systems in Japan from the HL7 version 2.5. These items were not included in the specifications of the SS-MIX2 and were not exported to SS-MIX2 standardized storage. We had to obtain these items from other data sources, such as the database of the EHR system, data warehouse, or receipt information.

MedicationRequest resources are displayed in the Medication Summary section instead of MedicationStatement resources. Data on medication requests could be obtained from SS-MIX2 but not from the medication statement data of a patient. In the recent FHIR US Core Profile, the MedicationStatement was replaced with MedicationRequest, while the IPS specification continued using the MedicationStatement. However, because the IPS specification uses open slices, MedicationRequest can also be used [8]. Although it is possible to estimate a patient’s medication intake status from past medication information to some extent, if the patient purchases and takes medication from a facility other than the EHR facility, the information will be incomplete. It would be necessary to accumulate data from Personal Health Records to obtain the complete Medication Summary dataset.

### Limitations

We confirmed some limitations of this research, not only regarding the implementation of the patient summary application but also related to the standardization of HL7 and IPS. Narrative items of unstructured text data, such as Inpatient Reasons, Physical Findings, Nursing Summary, and Discharge Status, were not included in the patient summary application. These four items are not required in the IPS but are required in the Japanese discharge summary. In nursing summaries, important information such as the patient’s condition, test results, treatment, and prognosis during hospitalization are often summarized into narrative sentences. Although we can acquire partial information from several types of FHIR resources, there are no standard ways to summarize this information. Furthermore, the mapping logic would become complex and impractical. There is a limitation in the current approach for extracting and summarizing important information from a large amount of unstructured data. A data-driven approach, such as natural language processing, should be introduced to summarize these items [21].

Current Problems and the History of Past Problems are displayed in the Problem List. In the case of a patient with a long history, physicians must seek the most important and related problems on the list. Narrowing down and extracting the important problems are necessary for efficient work. Data-driven approaches such as machine learning help narrow down problems with high relevance and importance in a long list [22].

Since the preprocessing and data conversion processes were performed in the previous study [12], the application in this study is available with the basic functions supported by the FHIR server. However, the lack of a module for additional data preprocessing could potentially limit the extent and complexity of queries that can be executed, particularly when dealing with large datasets or more complex queries.

This study focused on the minimum necessary patient information and standardized methods for summarizing patient information. Therefore, we used dummy data, including full information items, instead of actual patient data. In addition, we evaluated the application by simulating Japanese common use cases and comments from physicians instead of a UX test in the field. We then confirmed the feasibility and issues to be addressed. However, for practical use, it is necessary to conduct more rigorous UX tests, such as efficiency, effectiveness, and satisfaction by actual users, in future research.

### Future Work

We want to continue with research on the above issues confirmed in this research, adopting a data-driven approach to improve summarization accuracy and including real-user UX testing to validate the possibility for practical use. When these issues are resolved, a patient summary application based on standardized specifications will be practically realized in Japan. As a result, reducing the information search burden and overload would mitigate physicians’ burnout.

## Conclusions

This study implemented a patient summary application concerning IPS using FHIR resources converted from existing SS-MIX2 standardized storage. By evaluating the application in common use cases in Japan, we confirmed the feasibility and issues for future work. After resolving these issues, such a system will be practical in Japan for connecting and sharing patient information effectively between hospitals and clinics. Furthermore, this system is expected to reduce information overload, workload, and burnout.

## Data Availability

Data sharing is not applicable to this article as no datasets were generated or analyzed during the current study.
